# Simultaneous Analysis of Bergapten and Schinifoline in *Zanthoxylum schinifolium* Seeds Using HPLC and UPLC-MS/MS Systems

**DOI:** 10.3390/foods12071355

**Published:** 2023-03-23

**Authors:** Chang-Seob Seo

**Affiliations:** KM Science Research Division, Korea Institute of Oriental Medicine, Daejeon 34054, Republic of Korea; csseo0914@kiom.re.kr; Tel.: +82-42-868-9361

**Keywords:** simultaneous analysis, *Zanthoxylum schinifolium*, bergapten, schinifoline

## Abstract

*Zanthoxylum schinifolium* Siebold et Zuccarini belongs to the Rutaceae family and has been widely used as a spice in East Asian countries such as Korea, China, and Japan. The present study focused on developing and validating a simultaneous analytical method for marker substances (bergapten and schinifoline) in *Z. schinifolium* seeds. This was achieved using high-performance liquid chromatography with a photo-diode array detector (HPLC-PDA) and ultra-performance liquid chromatography with tandem mass spectrometry (UPLC-MS/MS) systems. In the regression equation, all markers showed a coefficient of determination of ≥0.9990. Marker recovery was 96.90–105.16% (relative standard deviation (RSD) ≤ 2.23), and the intra- and interday precision was RSD < 3.00. Bergapten and schinifoline were detected in the seeds at 1.70–2.85 mg/g and 0.19–0.94 mg/g, respectively. This analytical method will improve quality control of *Z. schinifolium* seeds. Additionally, this assay will provide basic data and quality assurance for future biological activity experiments or clinical applications.

## 1. Introduction

*Zanthoxylum schinifolium* Siebold et Zuccarini is a perennial plant belonging to the Rutaceae family and has been used as a spice in East Asian countries, including Korea, China, and Japan [1]. The *Zanthoxylum* genus consists of more than 200 *Zanthoxylum* species, among which *Z. piperitum* De Candolle, *Z. schinifolium* Siebold et Zuccarini, and *Z. bungeanum* Maximowicz are recorded in the Korean Pharmacopoeia [2,3,4].

A wide variety of phytochemical components such as coumarins (bergapten and umbelliferon) [5,6]; alkaloids (skimmianine, schinifoline, and sanshools) [3,5,6,7]; essential oils (limonene and linalool) [2,6]; fatty acids (oleic acid and palmitic acid) [2,6]; and glycosides (betulalbuside A and osmanthuside H) [7] have been isolated from these species.

Anti-inflammatory [2,6], antimicrobial [2], anticancer [8], analgesic [2,9], hepatoprotective [2,10], and antiviral [2,11] effects have been reported in studies of the *Zanthoxylum* species.

In the present study, an analytical method was developed for the simultaneous analysis of coumarin (bergapten), and an alkaloid (schinifoline) isolated from these *Zanthoxylum* species in *Z. schinifolium* seeds. Bergapten, a coumarin derivative, and schinifoline, a 4-quinolinone derivative, were first isolated from the leaves and pericarps of *Z. schinifolium*, respectively [5,12,13]. Bergapten has been reported to be effective in various areas such as neuroprotection, organ protection, anticancer, anti-inflammatory, antibacterial, and antidiabetic effects [14]. In comparison, schinifoline has been reported to have a radiosensitizing effect on human non-small cell lung cancer A549 cells [8] and an antifungal effect on *Candida albicans* [15].

Several studies on the analysis of Zanthoxylum species have been reported [2,3,9]. Li et al. [2] and Wu and Wu [9] analyzed the essential oils of *Z. myriacanthum* and *Z. schinifolium* using gas chromatography with mass spectrometry. In addition, Zhao et al. [3] quantitatively analyzed components of alkylamides, which are fragrance components, from *Z. schinifolium* oil using liquid chromatography with mass spectrometry. However, to our knowledge, analytical methods for bergapten and schinifoline in *Z. schinifolium* seeds have not yet been reported.

Therefore, in the present study, we endeavored to develop a simultaneous analysis method for bergapten and schinifoline in *Z. schinifolium* seeds using high-performance liquid chromatography with a photo-diode array detector (HPLC-PDA) and ultra-performance liquid chromatography with tandem mass spectrometry (UPLC-MS/MS).

## 2. Results

### 2.1. HPLC-PDA Analysis

#### 2.1.1. Selection of HPLC-PDA Analysis Conditions for Simultaneous Quantification

**Figure 1:**
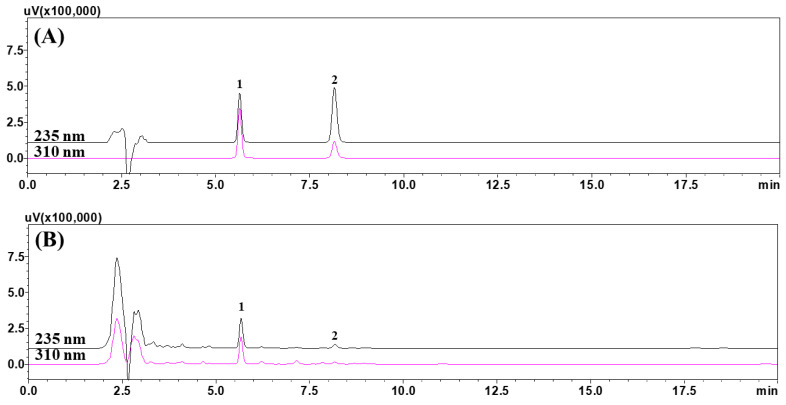

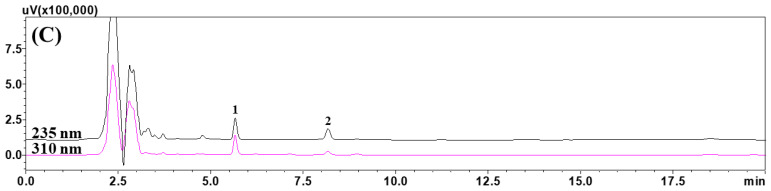
HPLC chromatograms of (**A**) a standard solution of the two marker substances, bergapten and schinifoline, (**B**) 70% ethanol extract of *Zanthoxylum schinifolium* seeds (2018ZSS), and (**C**) 70% ethanol extract of *Zanthoxylum schinifolium* seeds (2021ZSS). Bergapten (1) and schinifoline (2). The measured concentrations of the bergapten and the schinifoline in the standard solution (**A**) were each 50 μg/mL.

For quantitative analysis of bergapten and schinifoline in *Z. schinifolium* seeds, we used a Waters XBridge reverse-phase C_18_ column (250 length × 4.6 mm ID, 5 μm particle size) (Milford, MA, USA) with isocratic elution by mobile phases of a 1.0% (*v*/*v*) distilled water solution of acetic acid and 1.0% (*v*/*v*) solution of acetic acid in acetonitrile in a 1:1 ratio. Both marker components were monitored simultaneously at 235 nm (schinifoline) and 310 nm (bergapten) using a PDA. The flow rate was 1.0 mL/min, the injection amount was 10 μL during HPLC analysis. During the analysis, the column was maintained at 40 °C (Table S1 (Supplementary Materials)). A representative chromatogram is shown in Figure 1. The two markers, bergapten and schinifoline, were eluted with a resolution of ≥1.50 within 10 min (5.63 min and 8.15 min, respectively).

#### 2.1.2. Validation of the HPLC-PDA Analytical Method

Table 1 shows the results of parameters used to verify the analytical method we developed. In the calibration curve regression equation for bergapten and schinifoline, the coefficients of determination (*r*^2^) were both 0.9999 (Table 1 and Figure S1). Linearity of these two calibration curves was evaluated through the application of a Shapiro–Wilk test using the SigmaPlot 12.5 software (Systat Software, Inc., San Jose, CA, USA). As a result of the Shapiro–Wilk test, the *p*-value of both analytes was >0.05 at the 95% confidence level, indicating that the linear regression model had a normal distribution of the experimental data (Table 1). In addition, residuals of two markers show <2.0% (Figure S1). In the Shapiro–Wilk test for the residual distribution of the bergapten and schinifoline, the *p* values were 0.31 and 0.71, respectively, indicating that the data were normally distributed. The selectivity of each marker substance was confirmed by the UV spectrum of each compound (Figure S2) and the peak purity in the sample (Figure S3). The peak purity index values of bergapten and schinifoline were 1.0000, showing excellent selectivity, and impurities were not detected at each detection wavelength (235 nm for schinifoline and 310 nm for bergapten). The limit of detection (LOD) and the limit of quantitation (LOQ) of the two markers were 0.04–0.08 μg/mL and 0.12–0.24 μg/mL, respectively (Table 1). As shown in Table 2, it was validated by a standard addition method in which three different concentrations (low, medium, and high) were added. After adding the standard solution of each marker to the sample, ultrasonic extraction was performed for 30 min, followed by HPLC analysis. Both marker substances were measured at 96.02–100.96%, and the relative standard deviation (RSD) was calculated as ≤0.66 (Table 2). In intra- and interday precision tests for bergapten and schinifoline, RSD values were <0.40 (Table 3). In addition, the RSD value was <0.21% for the repeatability of retention time and peak area (Table S2). These results demonstrate that the precision of the developed assay is excellent.

#### 2.1.3. The Stability of the Markers

The stability of the two markers (bergapten and schinifoline) was measured at room temperature and under refrigeration (about 4 °C) for 10 days in standard and sample solutions, respectively. As shown in Table S3, the stability of the two markers was tested for 10 days (0, 1, 2, 3, 4, 7, and 10 days) at room temperature and under 4 °C refrigeration, respectively. As a result of comparing the bergapten and schinifoline for 10 days based on the first day (Day 0), 97.98–102.72% (RSD ≤ 1.55) and 98.30–103.99% (RSD ≤ 1.63) were measured, respectively. In the sample solution, the stability of the bergapten and schinifoline was measured as 97.58–100.09% (RSD < 1.00) and 99.08–100.52% (RSD < 1.00) compared to the initial data (Day 0) for 10 days at room temperature and under 4 °C refrigeration, respectively. We found that the two markers were stable for at least 10 days under either storage condition.

#### 2.1.4. Simultaneous Quantitation of Two Markers in *Z. schinifolium* Seeds

The assay developed in the present study was efficiently applied to the quantitative analysis of two markers in *Z. schinifolium* seeds. In the optimized HPLC analytical method, bergapten and schinifoline were detected at 2.30–2.85 mg/g and 0.33–0.94 mg/g, respectively, in 70% ethanol extracts from samples collected in 2018 (2018ZSS) and 2021 (ZSS2021) (Table 4).

### 2.2. UPLC-MS/MS Quantitation

#### 2.2.1. Setting Optimal UPLC-MS/MS Analytical Conditions for Quantitation

**Figure 2:**
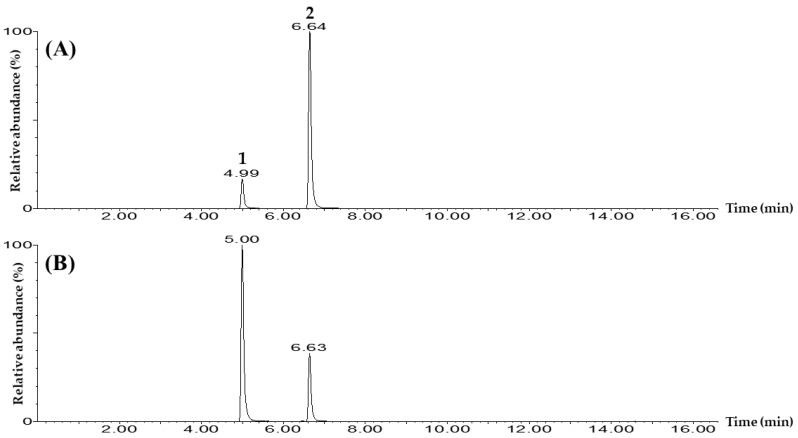
Total ion chromatography of (**A**) mixtures of the two markers, and (**B**) a 70% ethanolic extract of *Z. schinifolium* seeds measured by the UPLC-MS/MS MRM method in positive ion mode. Bergapten (1) and schinifoline (2). The measured concentrations of the bergapten and the schinifoline in the standard solution were both 0.5 μg/mL.

Quantitation of the two markers, bergapten and schinifoline, in *Z. schinifolium* seeds was performed using a UPLC-MS/MS (Waters, Milford, MA, USA) consisting of a Waters Acquity UPLC I-Class system and a tandem quadrupole MS detector system with an electrospray ionization source. Based on the analytical conditions presented in Table S4, bergapten and schinifoline were quantified by applying a multiple-reaction monitoring (MRM) method (Table 5). Bergapten and schinifoline were detected at 5.00 min and 6.64 min, respectively, in the form of [M + H]^+^ in positive ion mode (Figure 2 and Figure S4). To quantify the two markers, the precursor ion (Q1) and product ion (Q3) were set as follows [16,17,18,19]: Q3 of bergapten was set at m/z 202.0 in the form of [M + H−CH_3_]^+^ in which the methyl group was removed from Q1, and Q3 of schinifoline was set at m/z 173.1 in the form of [M + H−C_6_H_13_]^+^ in which the C_6_H_13_ functional group was removed from Q1 (Table 5, and Figures S5 and S6).

#### 2.2.2. Validation of the UPLC-MS/MS MRM Analytical Method

For the quantitation of bergapten, the regression equation for the calibration curve prepared at the concentrations of 0.10–5.00 μg/mL was *y* = 128,424.87*x* + 12,393.30 (*r*^2^ = 0.9990). In the case of schinifoline, the regression equation at the concentrations of 0.10–5.00 μg/mL was *y* = 520,305.56*x* + 73,378.28 (*r*^2^ = 0.9994) (Figure S7). As a result of analyzing the calibration curves of bergapten and schinifoline with the Shapiro–Wilk test of SigmaPlot 12.5 software (Systat Software, Inc., San Jose, CA, USA), the *p*-values were 0.65 and 0.17, respectively. These values had a *p*-value greater than 0.05 at the 95% confidence level, indicating that the linear regression model had a normal distribution of the experimental data. The residual of two markers was <2.0% (Figure S7). In the Shapiro–Wilk test for the residual distribution of the bergapten and schinifoline, the *p*-values were 0.18 and 0.30, respectively, indicating that the data were normally distributed. LOD and LOQ of the two markers were 0.01–0.06 (×10^−2^) μg/mL and 0.04–0.17 (×10^−2^) μg/mL, respectively. The recovery in this developed assay was validated by a standard addition method. As shown in Table 6, standard solutions of three different concentrations were added. UPLC-MS/MS analysis was performed after consecutive pretreatment of 5 min of ultrasonic extraction and 1 min of vortexing. Recovery of bergapten and schinifoline was measured at 99.63–105.16% (RSD ≤ 2.23, Table 6). The RSD value showing intra- and interday precision was less than 3.00% (Table 7). Additionally, the RSD value of repeatability for retention time and peak area was less than 0.50% (Table 7).

#### 2.2.3. Quantitation of Bergapten and Schinifoline in *Z. schinifolium* Seeds

The developed UPLC-MS/MS MRM method was successfully applied to the simultaneous quantification of bergapten and schinifoline in *Z. schinifolium* seeds. Bergapten was detected at 1.70–2.85 mg/g, and schinifoline was 0.19–0.90 mg/g (Table 8).

## 3. Discussion

In the present study, an analytical method for the simultaneous analysis of bergapten and schinifoline from *Z. schinifolium* seeds using HPLC and UPLC-MS/MS was developed and verified. Various constituents have been isolated and reported from *Zanthoxylum* species [2,3,5,6,7]. In particular, coumarins [5,6], alkaloids [3,5,6,7], and essential oils [2,6] have been reported.

Currently, HPLC is one of the most widely used analytical methods for natural product research in academia and industry, and it has the advantage of being relatively simple and easy to operate. However, UPLC-MS/MS is a more sensitive and accurate analytical method, and its use is increasing.

We conducted analyses to develop a quantitative method for the seeds of *Z. schinifolium* among several *Zanthoxylum* species using HPLC and UPLC-MS/MS systems. First, a simultaneous analytical method for bergapten and schinifoline was developed using an HPLC system. This method was validated through selectivity, linearity, recovery, and precision. Several types of reverse-phase C_18_ columns (SunFire, XBridge, and XTerr columns, 4.6 ID × 250 mm length, 5 μm particle size); acids (0.1% formic acid, 0.1% trifluoroacetic acid, 0.1% phosphoric acid, and 1.0% acetic acid); and column oven temperatures (30, 35, 40, and 45 °C) were tested for method development. When using a Waters XBridge reverse-phase C_18_ column maintained at 40 °C with isocratic elution by a distilled water-acetonitrile (both containing 1.0% acetic acid) mobile phase system, both markers were eluted within 10 min with a resolution of 12.15. Second, an analytical method for the two markers was developed using UPLC-MS/MS MRM with a Waters Acquity UPLC I-Class system and tandem quadrupole MS system. The assay developed by the two systems was validated through parameters such as selectivity, linearity, LOD, LOQ, recovery, and precision.

Based on the optimized HPLC and UPLC-MS/MS analytical methods described above, bergapten and schinifoline were successfully separated and quantitatively analyzed in two samples, 2018ZSS and 2021ZSS. In both assays, bergapten was more abundant than schinifoline. When comparing the content of both collection years, bergapten was more abundant in 2018ZSS than in 2021ZSS, while schinifoline was more abundant in 2021ZSS. The pattern was the same for both the HPLC and the UPLC-MS/MS analytical methods.

As shown in the results of this study, the UPLC-MS/MS method showed the advantages of shorter time, higher sensitivity, and separation with less solvent compared to the HPLC method. Nevertheless, the analysis method using HPLC, which is the most widely used and easy to operate to date, is considered a more appropriate analysis method for quality control of the two marker substances in *Z. schinifolium* seeds.

## 4. Materials and Methods

### 4.1. Chemicals and Reagents

**Figure 3:**
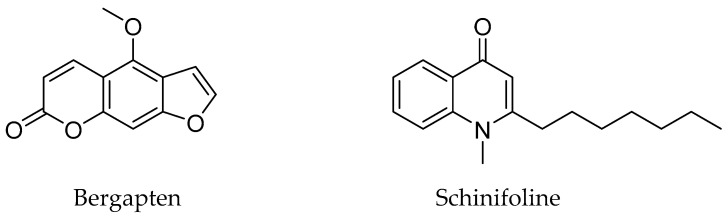
Chemical structures of the two markers selected of *Z. schinifolium* seeds.

The reference standard compounds for simultaneous analysis (Figure 3), bergapten (CAS No. 484-20-8, 98.0%, CFN98766) and schinifoline (CAS No. 80554-58-1, 99.1%, TBZ0836), were purchased from ChemFaces Biochemical Co. (Wuhan, China) and ChemNorm Biotech Co. (Wuhan, China), respectively. Solvents, methanol, acetonitrile, and distilled water were used at HPLC grade or LC–MS grade and were purchased from JT Baker (Phillipsburg, NJ, USA). Acetic acid (≥99.7%, A35-500) and formic acid (≥99.7%, A117-50) were HPLC and LC-MS grades, respectively, and these were purchased from Fisher Scientific (Fair Lawn, NJ, USA).

### 4.2. Plant Materials and Preparation of the 70% Ethanolic Extract of Z. schinifolium Seeds

Dried *Z. schinifolium* seeds (sample numbers 2018ZSS and 2021ZSS) were purchased from the *Z. schinifolium* producer Woobosancho (Miryang, Republic of Korea) in 2018 and 2021, respectively. The scientific name of the sample was confirmed in The Plant List (www.theplantlist.org) [20] and morphologically identified by Dr Goya Choi, Korea Institute of Oriental Medicine (Naju, Republic of Korea). Extracts of *Z. schinifolium* seeds were prepared by KOC Biotech Co. (Daejeon, Republic of Korea). In brief, 2.0 kg of the dried *Z. schinifolium* seeds, which were removed from their seedcase, were extracted under reflux at 80 °C for 3 h using 20 L of 70% ethanol. After filtering the extract using a standard sieve (270 mesh), the organic solvent was removed using a rotary evaporator. Then, the residue was suspended in 1.0 L of distilled water and made into a powdered sample (about 3.7%) using a freeze-dryer (LP20, Daejeon, Republic of Korea).

### 4.3. Preparation of Standard Stock and Sample Solutions

Standard stock solutions for simultaneous analysis of bergapten and schinifoline in *Z. schinifolium* seeds were prepared in methanol at 1000 ppm and stored under refrigeration (approximately 4 °C) until use. Subsequently, each prepared solution was serially diluted before use. After accurately taking 100 mg of the 70% ethanol extract of the two markers prepared for quantitative analysis using HPLC from *Z. schinifolium* seeds, 10 mL of 70% methanol was added and extraction was conducted ultrasonically for 30 min. Then, the sample solution for UPLC-MS/MS analysis was separately prepared with 70% methanol at a concentration of 50 mg/10 mL, followed by ultrasonic extraction for 5 min and vortexing for 1 min. All the extracted solutions were used for quantitative analysis after 0.2 μm-membrane filtration (Pall Life Sciences, Ann Arbor, MI, USA).

### 4.4. HPLC-PDA Analytical Conditions to Quantify Bergapten and Schinifoline in Z. schinifolium Seeds

Simultaneous determination of two markers, a coumarin derivative and an alkaloid (bergapten and schinifoline), respectively, was performed using a Shimadzu Prominence LC-20A series system coupled to a PDA detector (Kyoto, Japan). This system was controlled by LabSolution software (version 5.54, SP3, Kyoto, Japan). Detailed analytical conditions such as column, column temperature, mobile phase, and elution conditions are presented in Table S1. Eluate was monitored at 235 nm and 310 nm considering the UV absorption maxima of the target components.

### 4.5. UPLC-MS/MS Analytical Conditions to Quantify Bergapten and Schinifoline in Z. schinifolium Seeds

The quantitation of bergapten and schinifoline in *Z. schinifolium* seeds was archived using a UPLC-MS/MS system consisting of an Acquity UPLC system and a tandem triple quadrupole MS system (Waters, Milford, MA, USA). In the UPLC-MS/MS analysis, an electrospray ionization source was used as an ion source, and MassLynx software (version 4.2, Milford, MA, USA) was used for data acquisition and processing. Detailed UPLC and MS operating conditions for the quantitation are presented in Table S4, and UPLC-MS/MS MRM parameters are shown in Table 5.

### 4.6. Validation of the Developed Two Analytical Methods

The developed HPLC-PDA and UPLC-MS/MS analytical methods were validated by testing selectivity, linearity, LOD, LOQ, recovery, and precision.

First, its selectivity was determined by comparing the UV spectra of the markers in the standards and samples. In addition, the peak purity of the two components was evaluated in the sample solution. This was only verified using the HPLC-PDA method.

Second, the linearity of each marker was validated through the *r*^2^ calculated from the regression equation of the calibration curve measured triplicate. In addition, LOD and LOQ were calculated using the following Equations (1) and (2).

(1) $$\mathrm{LOD}\;\left(\mu g/\mathrm{mL}\right)=3.3\times \frac{\sigma }{S}\;\mathrm{and}$$

(2) $$\mathrm{LOQ}\;\left(\mu g/\mathrm{mL}\right)=10\times \frac{\sigma }{S}$$

where *σ* is the SD of the *y*-intercept in the regression equation for each marker, and *S* is the slope of the regression equation.

Third, the recovery of each marker was determined by the standard addition method. In brief, after accurately taking 100 mg of the sample, each of the two markers was added at three concentrations, prepared at 10 mg/mL using 70% methanol, and HPLC analysis was performed. The recovery (%) was calculated using the following Equation (3).

(3) $$\mathrm{Recovery}\;\left(\%\right)=\frac{\mathrm{Found}\;\mathrm{concentration}}{\mathrm{Spiked}\;\mathrm{concentration}}\times 100$$

Finally, precision was demonstrated using RSD (%) values. Intra- and interday precision were measured on day 1 and for three consecutive days, and then the RSD values were calculated. For repeatability, RSD values were calculated after six repeated measurements using a standard solution in which the two markers were mixed. The RSD (%) value was calculated as in Equation (4).

(4) $$\mathrm{RSD}\;\left(\%\right)=\frac{\mathrm{SD}}{\mathrm{Mean}}\times 100$$

### 4.7. Stability Test

The stability of bergapten (6.25 μg/mL) and schinifoline (1.25 μg/mL) was tested for 10 days (0, 1, 2, 3, 4, 7, and 10 days) at room temperature (23 ± 1 °C) and under refrigeration (approximately 4 °C) using standard solution. In addition, the stability of the two components was tested for 10 days using the sample solution prepared at a concentration of 10 mg/mL.

### 4.8. Statistical Analysis

All data used in this study were expressed as mean, SD, and RSD (%) using Microsoft Excel 2021 software (Microsoft, Redmond, WA, USA). Statistical analysis was performed using SigmaPlot software 12.5 (Systat Software, Inc., San Jose, CA, USA).

## 5. Conclusions

In the present study, bergapten and schinifoline were selected as marker substances for *Z. schinifolium* seeds, and an analytical method for the simultaneous analysis of these two markers from *Z. schinifolium* seeds was developed and validated for the first time, to our knowledge. This analytical method can be used as a basis for quality control of *Z. schinifolium* seeds and other herbal medicines. Furthermore, the method can be used to obtain basic data for biological activity research or clinical applications.

## Figures and Tables

**Table 1:** Linear range, regression equation, coefficients of determination (*r*^2^), limit of detection (LOD), and limit of quantitation (LOQ) for simultaneous quantitation of two marker substances in *Z. schinifolium* seeds.

Marker	Linear Range (μg/mL)	Regression Equation ^1^(*y* = a*x* + b)	*r* ^2^	*p*-Value ^2^	LOD (μg/mL) ^3^	LOQ (μg/mL) ^4^
Bergapten	1.56–100.00	*y* = 42,108.21*x* + 24,433.44	0.9999	0.30	0.08	0.24
Schinifoline	0.31–20.00	*y* = 71,671.58*x* + 7668.61	0.9999	0.28	0.04	0.12

^1^*y*: peak area of each marker substance, *x*: concentration of each marker substance. ^2^ *p* value of Shapiro–Wilk test (confidence level at 95%). ^3^ LOD: limit of detection. ^4^ LOQ: limit of quantitation.

**Table 2:** Results of recovery test of two marker substances in developed HPLC analytical method (*n* = 5).

Marker	Concentration (μg/mL)		Recovery (%)	SD ^1^	RSD (%) ^2^
	Spiked	Found			
Bergapten	5.00	4.94	98.73	0.27	0.27
	12.50	12.42	99.33	0.25	0.25
	25.00	24.93	99.74	0.07	0.07
Schinifoline	2.00	1.92	96.02	0.64	0.66
	4.00	4.04	100.96	0.35	0.35
	8.00	7.75	96.90	0.12	0.12

^1^ SD: standard deviation. ^2^ RSD: relative standard deviation.

**Table 3:** The precision of the HPLC analytical method of the two markers.

Marker Substance	Conc. (μg/mL)	Intraday (*n* = 5)			Interday (*n* = 5)		
		Found Conc. (μg/mL)	Precision (RSD, %)	Accuracy (%)	Found Conc. (μg/mL)	Precision (RSD, %)	Accuracy (%)
Bergapten	25.00	25.42	0.05	101.69	25.43	0.22	101.71
	50.00	50.44	0.17	100.88	50.32	0.26	100.63
	100.00	100.04	0.09	100.04	100.17	0.39	100.17
Schinifoline	5.00	5.06	0.25	101.26	5.05	0.29	101.09
	10.00	10.03	0.27	100.26	10.00	0.29	100.02
	20.00	20.02	0.06	100.10	20.04	0.39	100.19

**Table 4:** Quantitation of bergapten and schinifoline in *Z. schinifolium* seeds using the optimized HPLC-PDA analytical method (*n* = 5).

Marker	2018ZSS			2021ZSS		
	Mean (mg/g)	SD × 10^−1^	RSD (%)	Mean (mg/g)	SD × 10^−1^	RSD (%)
Bergapten	2.85	0.13	0.44	2.30	0.12	0.54
Schinifoline	0.33	0.01	0.32	0.94	0.03	0.36

**Table 5:** Parameters for UPLC-MS/MS MRM analysis of bergapten and schinifoline.

Marker	Ion Mode	Molecular Weight	MRM Condition	Cone Voltage (V)	Collision Energy (Ev)
Bergapten	Positive	216.04	216.9 → 202.0	30	20
Schinifoline	Positive	257.18	258.4 → 173.1	30	30

**Table 6:** Results of recovery test of two marker substances in developed UPLC-MS/MS analytical method (*n* = 5).

Marker	Concentration (μg/mL)		Recovery (%)	SD	RSD (%)
	Spiked	Found			
Bergapten	0.40	0.40	99.63	2.23	2.23
	0.80	0.84	105.16	1.10	1.05
	1.60	1.66	104.02	1.39	1.34
Schinifoline	0.02	0.02	99.68	0.22	0.22
	0.04	0.41	102.02	0.10	0.10
	0.08	0.81	100.94	0.19	0.19

**Table 7:** The precision and repeatability of the UPLC-MS/MS analytical method of the two markers.

Marker	Conc. (μg/mL)	Intraday (*n* = 5)			Interday (*n* = 5)			Repeatability (*n* = 6)	
		Found Conc. (μg/mL)	Precision (RSD, %)	Accuracy (%)	Found Conc. (μg/mL)	Precision (RSD, %)	Accuracy (%)	RSD (%) of Retention Time	RSD (%) of Peak Area
Bergapten	0.40	0.37	2.83	93.11	0.39	2.82	98.48	0.11	0.41
	0.80	0.77	1.43	96.42	0.81	1.07	101.86		
	1.60	1.53	1.91	95.44	1.63	2.34	101.75		
Schinifoline	0.02	0.02	0.02	97.45	0.02	0.25	100.12	0.08	0.16
	0.04	0.04	0.07	97.14	0.04	0.10	100.87		
	0.08	0.08	0.02	94.89	0.08	0.21	100.39		

**Table 8:** Quantitation of bergapten and schinifoline in *Z. schinifolium* seeds by UPLC-MS/MS MRM (*n* = 5).

Marker	2018ZSS			2021ZSS		
	Mean (mg/g)	SD	RSD (%)	Mean (mg/g)	SD	RSD (%)
Bergapten	2.85	0.15	5.30	1.70	0.06	3.45
Schinifoline	0.19	0.01	4.83	0.90	0.05	5.34

## Data Availability

All data supporting the present study can be found in this article.

## Supplementary Materials

The following supporting information can be downloaded at: https://www.mdpi.com/article/10.3390/foods12071355/s1, Figure S1: Calibration curves (A) and residual plots (B) for evaluating the linearity of the two markers in the HPLC-PDA method; Figure S2: Validation of selectivity through UV spectra comparison; Figure S3: Peak purity evaluation to demonstrate the selectivity of two components in a sample; Figure S4: Extracted ion chromatograms for each standard marker (A), 2018ZSS sample (B), and 2021ZSS sample (C) measured by LC-MS/MS MRM mode; Figure S5: Fragmentation of the two markers by the UPLC-MS/MS MRM method; Figure S6: Precursor ion (Q1) and product ion (Q3) peaks of bergapten (A) and schinifoline (B) by the UPLC-MS/MS MRM method; Figure S7: Calibration curves (A) and residual plots (B) of the two markers in the UPLC-MS/MS MRM method; Table S1: Chromatographic parameters for simultaneous quantitation of the two markers in *Z. schinifolium* seeds by HPLC-PDA; Table S2: Repeatability of retention time and peak area of the two markers (*n* = 6); Table S3: Stability (%) of the two markers measured at room temperature and under refrigeration using standard and sample solutions; Table S4: Parameters for simultaneous quantitation of the two markers in *Z. schinifolium* seeds by the UPLC-MS/MS MRM method.

## References

[1] Oh M., Chung M.S. (2014). Effects of oils and essential oils from seeds of *Zanthoxylum schinifolium* against foodborne viral surrogates. Evid. Based Complement. Alternat. Med..

[2] Li R., Yang J., Shi Y., Zhao M., Ji K., Zhang P., Xu Y., Hu H. (2014). Chemical composition, antimicrobial and anti-inflammatory activities of the essential oil from Maqian (*Zanthoxylum myriacanthum* var. *pubescens*) in Xishuanghanna, SW China. J. Ethnopharmacol..

[3] Zhao Z.F., Zhu R.X., Zhong K., He Q., Luo A.M., Gao H. (2013). Characterization and comparison of the pungent components in commercial *Zanthoxylum bungeanum* oil and *Zanthoxylum schinifolium* oil. J. Food Sci..

[4] Chun I.K. (2019). The Korean Pharmacopoeia.

[5] Wang K., Meng X.H., Chai T., Wang C.B., Sang C.Y., Wang W.F., Shang X.Y., Yang J.L. (2021). Chemical constituents from the fruits of *Zanthoxylum bungeanum* and their chemotaxonomic significance. Biochem. Syst. Ecol..

[6] Negi J.S., Bisht V.K., Bhandari A.K., Singh P., Sundriyal R.C. (2011). Chemical constituents and biological activities of the genus *Zanthoxylum*: A review. Afr. J. Pure Appl. Chem..

[7] Li W., Yang S.Y., Yan X.T., Sun Y.N., Song S.B., Kang H.K., Kim Y.H. (2014). NF-kB inhibitory activities of glycosides and alkaloids from *Zanthoxylum schinifolium* stems. Chem. Pharm. Bull..

[8] Wang C.F., Fan L., Tian M., Qi X.S., Liu J.X., Feng J.B., Du S.S., Su X., Wang Y.Y. (2014). Radiosensitizing effect of schinifoline from *Zanthoxylum schinifolium* Sieb et Zucc on human non-small cell lung cancer A549 cells: A preliminary in vitro investigation. Molecules.

[9] Wu Y., Wu H. (2014). Analgesia synergism of essential oil from pericarp of Zanthoxylum schinifolium and verapamil. Evid. Based Complement. Alternat. Med..

[10] Lee S.J., Lim K.T. (2008). Glycoprotein of *Zanthoxylum piperitum* DC has a hepatoprotective effect via anti-oxidative character in vivo and in vitro. Toxicol. Vitr..

[11] Choi H.J. (2016). Evaluation of antiviral activity of *Zanthoxylum* species against picornaviruses. Osong Public Health Res. Perspect..

[12] Fang Z., Jun D.Y., Kim Y.H., Min B.S., Kim A.K., Woo M.H. (2010). Cytotoxic constituents from the leaves of *Zanthoxylum schinifolium*. Bull. Korean Chem. Soc..

[13] Liu S.L., Wei L.X., Wang D., Gao C.Y. (1991). Studies on the chemical constituents from the peel of *Zanthoxylum schinifolium* Sieb et Zucc. Acta Pharm. Sin..

[14] Liang Y., Xie L., Liu K., Cao Y., Dai X., Wang X., Lu J., Zhang X., Li X. (2021). Bergapten: A review of its pharmacology, pharmacokinetics, and toxicity. Phytother. Res..

[15] Lu L., Li Z., Shan C., Ma S., Nie W., Wang H., Chen G., Li S., Shu C. (2021). Whole transcriptome analysis of schinifoline treatment in *Caenorhabditis elegans* infected with *Candida albicans*. Mol. Immunol..

[16] Lehr G.J., Barry T.L., Franolic J.D., Petzinger G., Scheiner P. (2003). LC determination of impurities in methoxsalen drug substance: Isolation and identification of isopimpinellin as a major impurity by atmospheric pressure chemical ionization LC/MS and NMR. J. Pharm. Biomed. Anal..

[17] Tang D.Q., Zheng X.X., Chen X., Yang D.Z., Du Q. (2014). Quantitative and qualitative analysis of common peaks in chemical fingerprint of Yuanhu Zhitong tablet by HPLC-DAD–MS/MS. J. Pharm. Anal..

[18] Wang X.X., Zan K., Shi S.P., Zeng K.W., Jiang Y., Guan Y., Xiao C.L., Gao H.Y., Wu L.J., Tu P.F. (2013). Quinolone alkaloids with antibacterial and cytotoxic activities from the fruits of *Evodia rutaecarpa*. Fitoterapia.

[19] Su C.H., Cheng Y.C., Chang Y.C., Kung T.H., Chen Y.L., Lai K.H., Hsieh H.L., Chen C.Y., Hwang T.L., Yang Y.L. (2022). Untargeted LC-MS/MS-based multi-informative molecular networking for targeting the antiproliferative ingredients in *Tetradium ruticarpum* fruit. Molecules.

[20] The Plant List. www.theplantlist.org.

