# Supplementary material for: Simultaneous Analysis of Bergapten and Schinifoline in Zanthoxylum schinifolium Seeds Using HPLC and UPLC-MS/MS Systems
Source: Foods. 2023 Mar 23;12(7):1355. doi: 10.3390/foods12071355 (PMC10093454; doi:10.3390/foods12071355)
Supplement: Supplementary file 1 [file foods-12-01355-s001.zip › foods-2273132-Supplementary Material.pdf]

Table S1

Chromatographic parameters for simultaneous quantitation of the two markers in *Z. schinifolium* seeds by HPLC–PDA.

**Chromatographic parameter**	
Analytical column	XBridge reverse-phase C_{18} (250 mm length × 4.6 mm ID, 5 µm particle size)
Detector	PDA[1] (235 and 310 nm)
Flow rate	1.0 mL/min
Injection volume	10.0 µL
Column temperature	40.0 °C
Mobile phase	1.0% (v/v) distilled water solution of acetic acid : 1.0% (v/v) solution of acetic acid in acetonitrile (1 : 1)

[1] PDA: photo-diode array

Table S2

Repeatability of retention time and peak area of the two markers (n = 6).

No.	Retention time (min)		Peak area (mAU)	
	**Bergapten**	**Schinifoline**	**Bergapten**	**Schinifoline**
1	5.631	8.149	2,409,851	1,395,099
2	5.628	8.146	2,412,426	1,396,535
3	5.633	8.151	2,410,016	1,395,809
4	5.629	8.146	2,404,693	1,391,498
5	5.637	8.152	2,401,711	1,391,360
6	5.634	8.150	2,400,370	1,390,291
Mean	5.632	8.149	2,406,511.167	1,393,432.000
SD	0.003	0.003	4,949.441	2,681.680
RSD (%)	0.059	0.031	0.206	0.192

The concentration of the analyzed standard solution was both 50 µg/mL.

Table S3

Stability (%) of the two markers measured at room temperature and under refrigeration using standard and sample solutions.

Day	Standard solution[1]				Sample solution[2]			
	Room temperature[3]		Refrigeration[4]		Room temperature		Refrigeration	
	Bergapten	Schinifoline	Bergapten	Schinifoline	Bergapten	Schinifoline	Bergapten	Schinifoline
0	100.00	100.00	100.00	100.00	100.00	100.00	100.00	100.00
1	99.82	100.09	99.21	99.44	99.54	99.55	100.34	98.37
2	102.13	102.42	98.51	98.40	99.33	99.24	100.29	98.26
3	100.05	99.69	98.30	100.21	99.52	99.84	100.30	98.38
4	99.81	101.05	100.49	100.63	98.69	99.02	100.19	98.36
7	102.72	102.55	101.49	101.32	97.58	99.79	100.17	98.19
10	97.98	98.59	102.95	103.39	97.65	99.75	100.03	98.13
Mean	100.19	100.43	99.95	100.31	98.90	99.60	100.19	98.53
SD	1.55	1.46	1.63	1.54	0.96	0.35	0.13	0.66
RSD (%)	1.55	1.46	1.63	1.53	0.97	0.35	0.13	0.67

[1] Concentration of standard solution was 6.25 µg/mL (bergapten) and 1.25 µg/mL (schinifoline). [2] Concentration of sample solution was 10.0 mg/mL. [3] Room temperature: 23 ± 1 °C. [4] Refrigeration: approximately 4 °C.

Table S4

Parameters for simultaneous quantitation of the two markers in *Z. schinifolium* seeds by the UPLC–MS/MS MRM method.

<table>
<tr><th>UPLC conditions</th><th></th><th>MS conditions</th><th></th></tr>
<tr><td>UPLC system</td><td>Acquity UPLC I-Class</td><td>MS detector</td><td>TQD[1]</td></tr>
<tr><td>Column</td><td>Acquity UPLC BEH C_{18} analytical column (2.1 mm × 100 mm, 1.7 μm)</td><td>MS software</td><td>MassLynx v4.2</td></tr>
<tr><td>Column temp.</td><td>45 °C</td><td>Ion source</td><td>ESI[2] positive</td></tr>
<tr><td>Sample temp.</td><td>5 °C</td><td>Acquisition mode</td><td>MRM[3]</td></tr>
<tr><td>Injection volume</td><td>2.0 μL</td><td>Capillary voltage</td><td>3.0 kV</td></tr>
<tr><td>Flow rate</td><td>0.3 mL/min</td><td>Cone gas flow</td><td>50 L/h</td></tr>
<tr><td>Mobile phase A</td><td>0.1% (v/v) formic acid in distilled water</td><td>Desolvation gas flow</td><td>500 L/h</td></tr>
<tr><td>Mobile phase B</td><td>Acetonitrile</td><td>Desolvation temp.</td><td>300 °C</td></tr>
<tr><td>Gradient</td><td><table>
<tr><th>Time (min)</th><th>A (%)</th><th>B (%)</th></tr>
<tr><td>Initial</td><td>80</td><td>20</td></tr>
<tr><td>0.1</td><td>80</td><td>20</td></tr>
<tr><td>14.0</td><td>5</td><td>95</td></tr>
<tr><td>15.0</td><td>0</td><td>100</td></tr>
<tr><td>15.1</td><td>80</td><td>20</td></tr>
<tr><td>18.0</td><td>80</td><td>20</td></tr>
</table></td><td>Source temp.</td><td>120 °C</td></tr>
</table>

[1] TQD: triple quadrupole detector. [2] ESI: electrospray ionization. [3] MRM: multiple reaction monitoring.

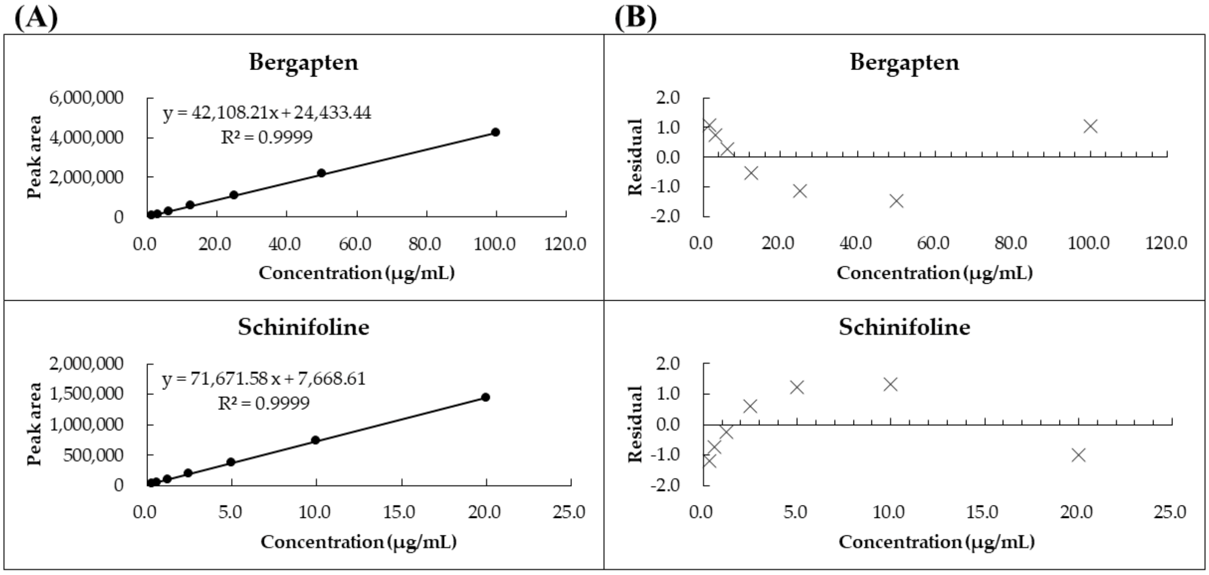

Figure S1. Calibration curves (A) and residual plots (B) for evaluating the linearity of the two marker substances in HPLC–PDA method.

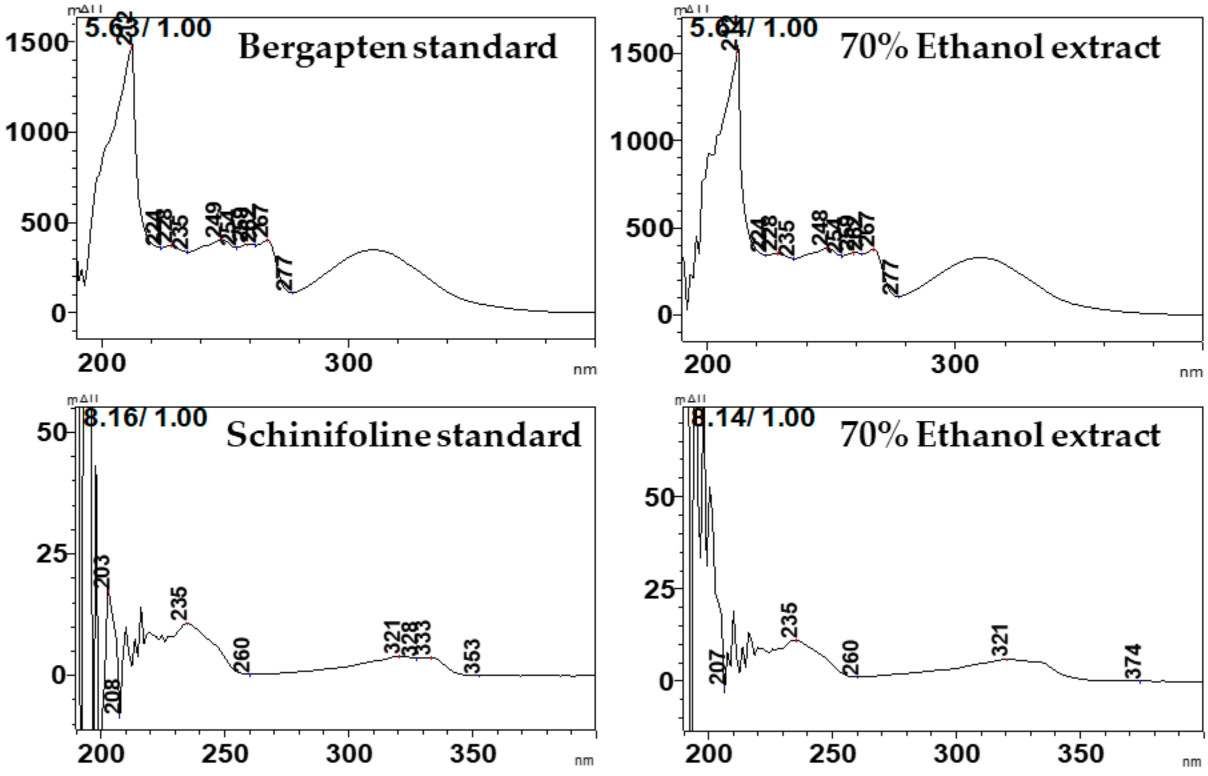

Figure S2. Validation of selectivity through UV spectra comparison.

Bergapten

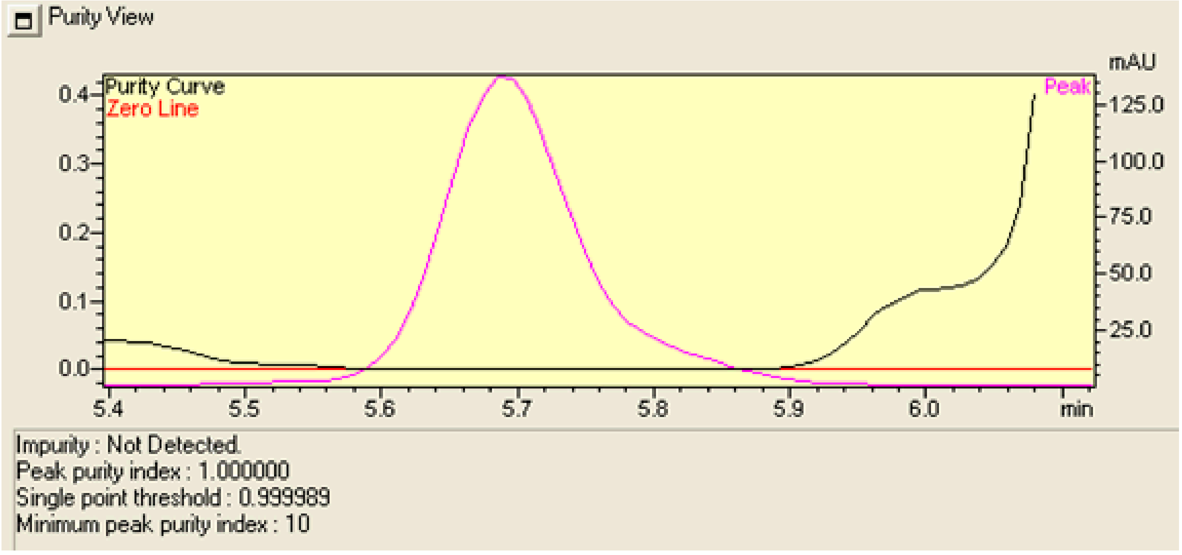

Schinifoline

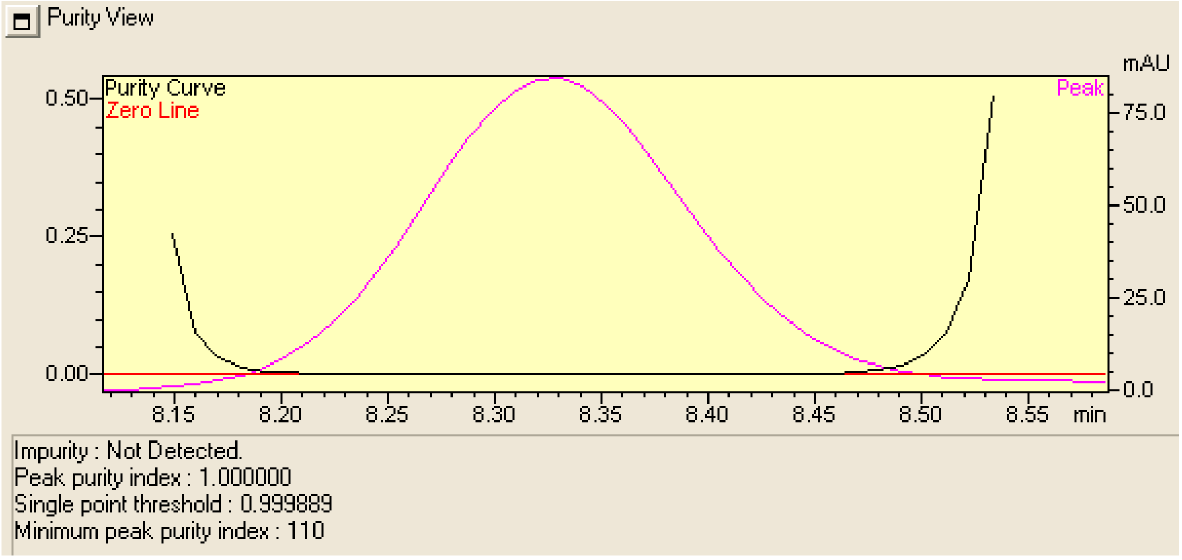

Figure S3. Peak purity evaluation to demonstrate the selectivity of two components in a sample.

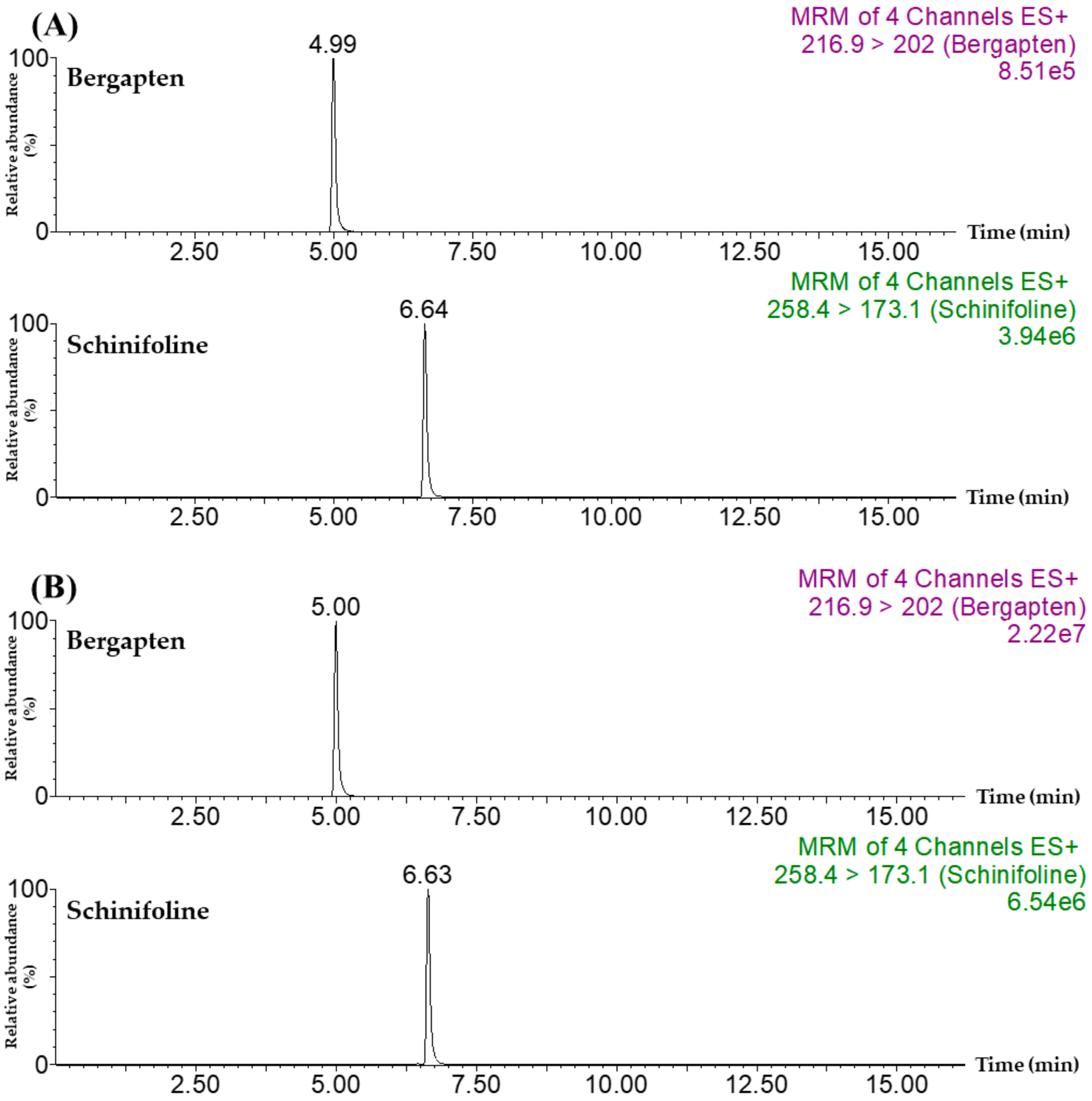
(A)
MRM of 4 Channels ES+
216.9 > 202 (Bergapten)
8.51e5
4.99
Bergapten
MRM of 4 Channels ES+
258.4 > 173.1 (Schinifoline)
3.94e6
6.64
Schinifoline
(B)
MRM of 4 Channels ES+
216.9 > 202 (Bergapten)
2.22e7
5.00
Bergapten
MRM of 4 Channels ES+
258.4 > 173.1 (Schinifoline)
6.54e6
6.63
Schinifoline
Relative abundance (%)
Time (min)
0
100
2.50
5.00
7.50
10.00
12.50
15.00

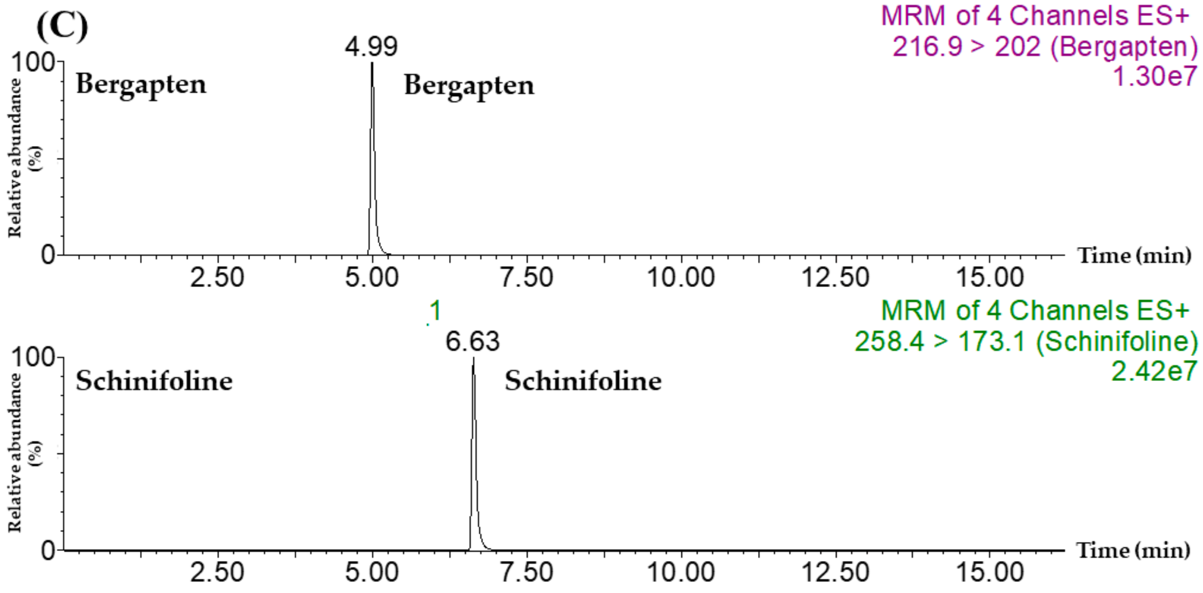

Figure S4. Extracted ion chromatograms for each standard marker (A), 2018ZSS sample (B), and 2021ZSS sample (C) measured by LC–MS/MS MRM mode.

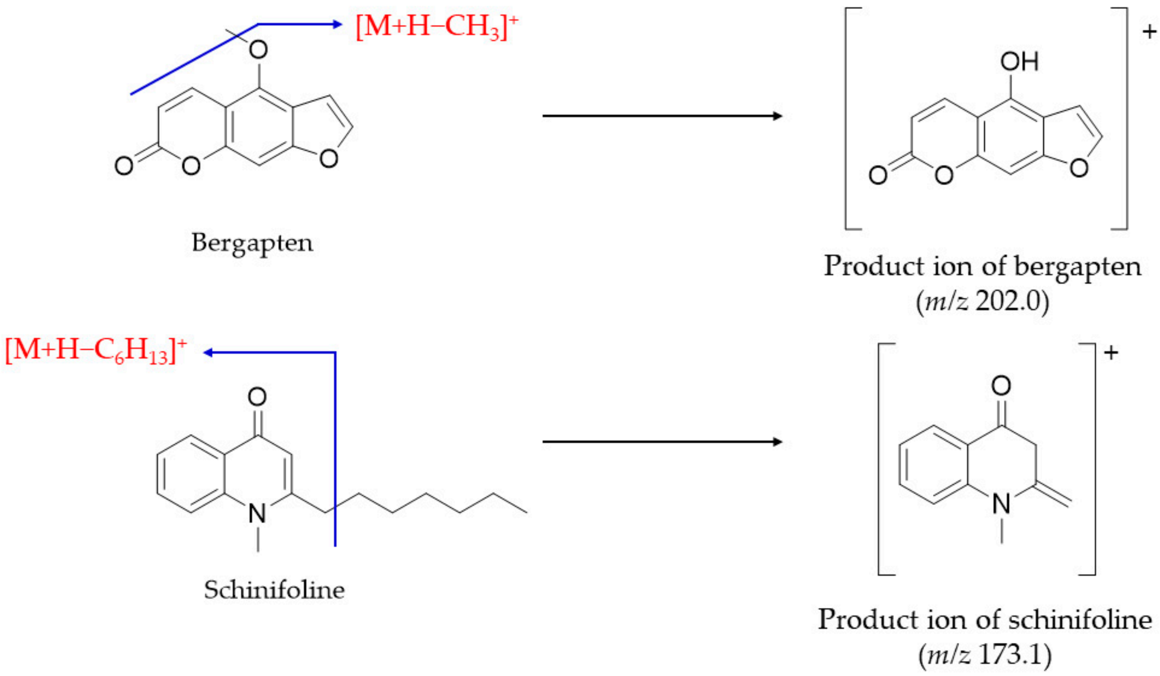

Figure S5. Fragmentation of the two markers by the UPLC–MS/MS MRM method.

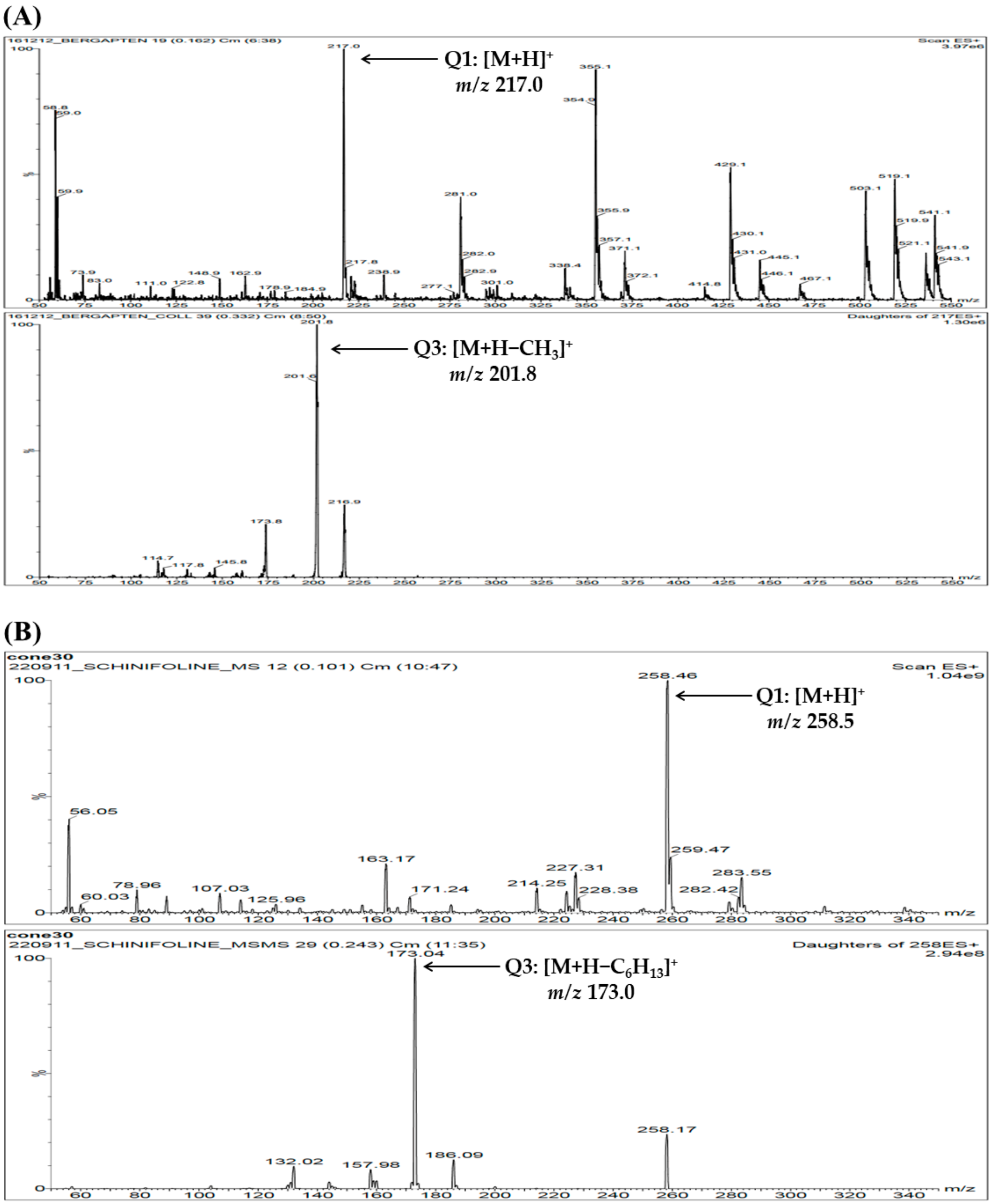

Figure S6. Precursor ion (Q1) and product ion (Q3) peaks of bergapten (A) and schinifoline (B) by the UPLC–MS/MS MRM method.

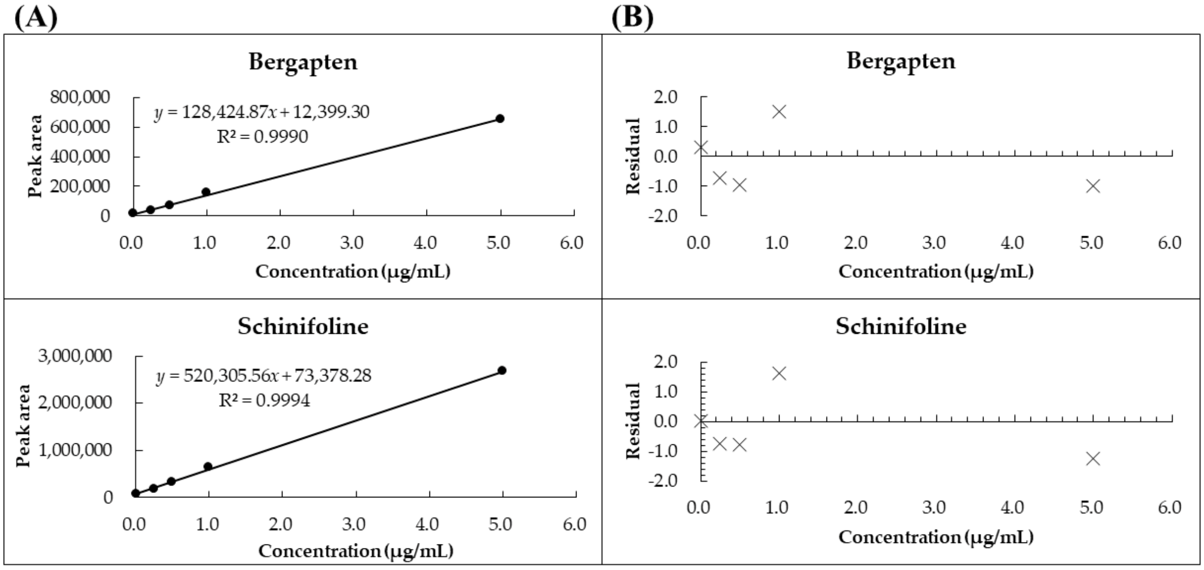

Figure S7. Calibration curves (A) and residual plots (B) of the two markers in UPLC–MS/MS MRM method.
